# Design, Content and Ecological Validity and Reliability of the Physical Activity and Sport Habits Questionnaire for Children Aged 8–12 Years in the Province of Gipuzkoa (Spain)

**DOI:** 10.3390/children12010100

**Published:** 2025-01-16

**Authors:** Aduna Badiola-Lekue, Irantzu Ibañez, Maite Fuentes, Javier Yanci, Oidui Usabiaga, Aitor Iturricastillo

**Affiliations:** 1 Research Group in Physical Activity, Physical Exercise and Sport (AKTIBOki) and Society, Sport and Physical Activity (GIKAFIT) Research Group, Department of Physical Education and Sports, Faculty of Education and Sport, University of the Basque Country (UPV/EHU), 01007 Vitoria-Gasteiz, Spain; aduna.badiola@ehu.eus (A.B.-L.); oidui.usabiaga@ehu.eus (O.U.); aitor.iturricastillo@ehu.eus (A.I.); 2 Research Group in Physical Activity, Physical Exercise and Sport (AKTIBOki) and Society, Sport and Physical Activity (GIKAFIT) Research Group, Department of Didactics of Musical, Plastic and Corporal Expression, Faculty of Education and Sport, University of the Basque Country (UPV/EHU), 01007 Vitoria-Gasteiz, Spain; irantzu.ibanez@ehu.eus; 3 Department of Physical Education and Sports, Faculty of Education and Sport, University of the Basque Country (UPV/EHU), 01007 Vitoria-Gasteiz, Spain; maite.fuentes@ehu.eus

**Keywords:** physical activity, sport habits, healthy habits, education, extracurricular sport, primary

## Abstract

**Background/Objectives:** This study aimed to develop a questionnaire to describe and diagnose the physical activity and sport (PAS) habits of 8–12-year-old schoolchildren, assessing its content, ecological validity and reliability, from a multidimensional perspective aligned with Global Matrix 4.0 indicators. **Methods:** The questionnaire design phase involved seven individuals from the university sector and sport managers from the Gipuzkoa Provincial Council. Seventeen experts later evaluated the questionnaire’s content and ecological validity. For reliability testing, 276 schoolchildren aged 8 to 12 completed the questionnaire twice, with a time interval of two weeks to two months. Statistical analyses included the Wilcoxon test to compare expert ratings, effect size and percentage change calculations for magnitude assessment, and McNemar, McN-Bowker or Wilcoxon tests to compare differences between initial and repeat responses. Cohen’s Kappa was used to assess agreement. **Results:** The initial battery of items, submitted to the validation process, comprised 31 items across 10 dimensions, derived from validated questionnaires and published works. Following content and ecological validity evaluations, modifications were made and nine items were removed due to improved wording, clarification of concepts, redundancy or lack of relevance. Expert quantitative analyses indicated improved overall questionnaire values. Reliability analysis revealed significant differences in five of the twenty-two items, though substantial agreement (from slight to almost perfect) was observed in twenty items. **Conclusions:** The study confirmed the questionnaire’s validity and reliability as a suitable tool for assessing PAS practices among 8–12-year-old schoolchildren in Gipuzkoa, Spain, in both Basque and Spanish languages.

## 1. Introduction

The multiple benefits produced by physical activity (PA) and sport (PAS) have been described in many previous studies [1,2,3]. It has been indicated that the regular practice of PAS had positive effects on physical and physiological health [4,5,6,7,8] and could also cause benefits in cognitive function and in different psychological aspects such as depression, anxiety, stress and self-concept [9,10,11]. It could also improve social skills, thus increasing general well-being [12,13,14,15], and it is considered fundamental in the transmission of social values and in comprehensive child personal development [16,17].

However, despite the increasing information and knowledge about the benefits of practising PAS, physical inactivity and sedentary lifestyle have become one of the main risk factors for non-communicable diseases, having a great impact on the world population of all ages [18,19,20]. Numerous studies and institutions have warned that children and young individuals performed little PA, which did not meet the recommendations provided by different institutions [21,22,23], and that sedentary habits had increased [24,25,26]. Furthermore, low levels of PA and/or physical exercise practice seemed to be even higher with increasing age, with increased abandonment of PAS, especially among adolescents [27,28,29,30,31]. It is known that an adequate level of PAS practice in childhood can cause greater adherence in adolescence and adulthood [31,32]. For this reason, childhood is a key period to inculcate practice habits and ensure that they are maintained in later stages [33,34]. In this sense, it is essential to understand the PAS habits of the child population from different areas and, likewise, count on valid and reliable instruments that allow these behaviours to be measured accurately.

In order to achieve an increase in the level of PAS practice in children, different action plans or strategies have been assessed and proposed in recent years [35,36,37,38]. Most of these action plans aimed at increasing the levels of PAS practice point to the need to approach the problem with a holistic vision [39,40] and from an ecological perspective in order to create active living communities [41,42,43]. In this sense, initiatives such as the Global Matrix on PA for children and adolescents have been developed to evaluate health promotion from a multidimensional perspective [44]. This initiative is based on understanding the main correlates of PA and employing knowledge translation models in health, ensuring effective and dynamic connections between research and practice. This proposal highlights the need to comprehensively address the understanding of the global variation in the level of PAS practice in children and adolescents through a unification of indicators related to PAS and the key sources of influence [45]. Among other aspects, by means of its indicators, Global Matrix 4.0 attempts to provide a holistic response to the PAS habits of children and adolescents around the world in a unified way. These indicators refer to the following: (1) overall PA; (2) organised sport and PA; (3) active play; (4) active transportation; (5) sedentary behaviour; (6) physical fitness; (7) family and peers; (8) school; (9) community and environment; and (10) government. However, most of the questionnaires related to the practice of PAS in children validated to date have only delved into some isolated and independent indicators presented in Global Matrix 4.0 [46,47,48,49,50,51,52,53,54,55,56,57].

Previously, questionnaires such as the “International Physical Activity Questionnaire” [58] have been widely used to measure the intensity, frequency per week and duration per day of PA. Different versions of the aforementioned questionnaire have been developed to adapt it to specific populations, such as the “PA Questionnaire for Adolescents” [59], which assesses the PA performed by the adolescent population in the previous seven days, or the “Physical Activity Questionnaire for children”, specific for children aged between eight and fourteen years [60].

Although these questionnaires have been widely used, most of them only focus on some specific dimensions of PAS practice; and as established in Global Matrix 4.0 [61], it is also important to collect information about active transportation and participation in organised sports and/or school sport programmes. For example, the “Assessment of Physical Activity Levels” questionnaire, designed by Telama et al. [62], includes items relating to organised PA, recreational PA and PA performed in physical education classes, in addition to analysing participation in sport competitions. On the other hand, studies have addressed the relevance of understanding the factors, beliefs and barriers that determine the levels of PA in children and adolescents [50], as well as the accessibility to spaces, facilities and infrastructures available to them. Moreover, the influence of the social environment on children’s PAS levels has also been assessed in isolation [63], and for the future, it is considered important to determine the PA state in which each person is with respect to PAS, to recognise the intention of practising PA and/or sport on a regular basis [64].

In this sense, there seems to be a gap in the scientific literature on the validity of questionnaires that address the different indicators of Global Matrix 4.0 as a whole. Therefore, the purpose of this study was to design a multidimensional PAS habits questionnaire for 8- to 12-year-old schoolchildren. The study also sought to establish its content and ecological validity, intending for it to serve as a tool for describing and diagnosing PAS habits. Ultimately, this instrument aims to inform public policies aimed at promoting PAS among children.

## 2. Materials and Methods

### 2.1. Design

The present study was conducted in two phases. In the first phase, the questionnaire was designed and the content and ecological validities were assessed by a panel of experts. In the second phase, the reliability of the questionnaire developed in the first phase was determined by administering it to children aged 8–12 years old twice: test (T) and re-test (R-T).

### 2.2. Participants

Twenty-four individuals participated in the first phase. Four researchers, all of them with PhDs in PA and sports sciences, with extensive academic and professional experience, and three sport managers from the Gipuzkoa Provincial Council designed the questionnaire. Subsequently, the questionnaire was reviewed in two assessment phases by a panel of seventeen experts (nine with a teaching and/or researcher profile, and eight coordinators of school sport programmes). The panel of nine teachers and/or researchers had to meet three of the five established criteria: university teacher, PhD, research in the area of school sports, at least ten years of professional experience in the area of school sports and expert in the two languages in which the questionnaire was created. Coordinators of school sport programmes had to meet the three established criteria: bachelor’s degree in PA and sports sciences, at least four years of professional experience in the area of school sports, and expert in the two languages in which the questionnaire was created.

In the second phase, initially 356 schoolchildren from 16 schools participated in the test and 298 in the re-test, of whom 276 completed the questionnaire twice and were included in the study. The sample was drawn by the research team according to the characteristics of the total population of the province under study (Gipuzkoa, Spain). Recruitment was carried out through the collaboration established between the Gipuzkoa Provincial Council and the management of the educational institutions. The distribution of the total sample according to gender was 48.2% girls, 50% boys and 1.8% preferred not to answer; no one identified as non-binary. With respect to age and school type, 46.7% were attending the third or fourth year of primary education and 53.3% the fifth or sixth year; of the total of schools (n = 16), 39.9% were public and 60.1% were concerted or private. Regarding the seven regions that compose the province, 8.7% belonged to region 1, 7.2% to region 2, 2.5% to region 3, 12.7% to region 4, 9.1% to region 5, 41.7% to region 6 and 18.1% to region 7.

All participants responded to the questionnaire voluntarily from 10 December 2021 to 8 April 2022 and were free to withdraw from the study at any time. Previously written informed consent was obtained from the parents or legal guardians of the schoolchildren, and the present study was approved by the Human Research Ethics Committee (CEISH, cod. M10_2021_252) of the University of the Basque Country (UPV/EHU) and followed the guidelines established in the Declaration of Helsinki [65].

### 2.3. Procedure

In the first phase, in order to perform an intervention based on evidence [66] and with the proven effectiveness of a participatory and co-creative process [67,68], a working group was established with four researchers from the research group who conducted a comprehensive review of the literature on the subject. The main dimensions established in Global Matrix 4.0 were identified, and relevant items from other questionnaires for each dimension were selected. Afterwards, three sport managers from the Gipuzkoa Provincial Council made contributions and modifications specific to the context of application, and the first draft of the multidimensional ad hoc questionnaire was prepared. The content of this first version was subjected to validation by a panel of experts composed of seventeen individuals, who reviewed the questionnaire in two phases. In phase one, the experts assessed each of the items in the questionnaire both qualitatively (general observations and translation) and quantitatively (1 = does not meet the criteria; 2 = low level; 3 = moderate level; 4 = high level). The following indicators were taken into consideration: sufficiency (the items that belong to the same dimension are enough to obtain its measurement); clarity (the item is easily understood, i.e., its syntax and semantics are adequate); coherence (the item has a logical relationship with the dimension being measured); and relevance (the item is essential or important, i.e., it should be included) [69].

Once the group of experts performed the qualitative and quantitative review of the questionnaire, all contributions were collected, and the corresponding modifications were made by the researchers. This way, a second version of the questionnaire was obtained. In the second phase of the revision, the experts scored each item only quantitatively. The three sport managers reviewed the different versions of the questionnaire, contrasting whether the entire questionnaire and each item in particular reflected the Gipuzkoa reality, thus determining its ecological validity.

In the second phase, the questionnaire was distributed among the students on two occasions, from two weeks to two months apart. Before distribution, the questionnaire was tested in a classroom with a small group of 15 students to ensure their understanding. The distribution period was scheduled based on the academic calendar and the school sports program, aligning with the most stable period for PAS practice. This timing was agreed upon with the Provincial Council of Gipuzkoa and the school administration. On both occasions, the questionnaire was completed online at the corresponding educational institutions and in the presence of the teachers. The procedure for completing the questionnaire was explained to all participants and, if they had any questions, the teachers had a help guide available.

### 2.4. Measuring Instrument

The questionnaire “Physical Activity and Sport Habits Questionnaire for Children” was designed to analyse PAS habits and measure the level of practice in schoolchildren aged from eight to twelve years. The goal was to meet a need detected by the Gipuzkoa Provincial Council related to knowing in depth the habits of Gipuzkoa schoolchildren in order to optimise PAS promotion strategies.

In phase one, 31 items that corresponded to validated questionnaires and previously published works were chosen and distributed into ten dimensions for the composition of an initial battery of possible items [46,47,48,49,50,51,52,53,54,55,56,57,70]. These dimensions included the relevant school-age indicators established by the Global Matrix 4.0 [61].

After submitting the initial 31-item questionnaire to the validation process, following the expert panel’s advice, nine items were eliminated and a questionnaire of twenty-two items distributed into nine dimensions was created. After the second reliability phase, the final version of the questionnaire (Appendix A and Appendix B) maintained the number of items (n = 22; 14 with a single response option, 6 with multiple response options and 2 with an open response) and dimensions (n = 9; general PA, transportation, recreational screen time, family and friends, spaces/facilities, sleep, barriers, motivations and current and future state of PA).

### 2.5. Statistical Analysis

The statistical analysis was performed using the Statistical Package for Social Sciences (SPSS Inc. version 28.0 Chicago, IL, USA), and the statistical significance was established at *p* < 0.05. Regarding the first phase, the results obtained by the experts are presented as mean ± standard deviation. Kolmogorov–Smirnov test was used to assess the normality of the data. The Wilcoxon test was performed to compare the differences in means between the quantitative values that the experts had reported in the first and second phases. The magnitude of the differences was calculated using the effect size in the Wilcoxon test and percentage changes (Δ%). The magnitude of the changes was interpreted as follows: no effect/very small = 0–0.09; small = 0.10–0.29; medium = 0.30–0.49; and large = >0.50 [71].

In the second phase, the differences between the answers given by the students in the T and the R-T were calculated using the McNemar, McN-Bowker or Wilcoxon tests for dichotomous qualitative values, qualitative values with more than two response options and ordinal values, respectively. Furthermore, Cohen’s Kappa test was used to assess the agreement between the results of the questionnaire in the T and the R-T. In the latter case, the interpretation of the values was performed using the classification system proposed by Landis and Koch [72], namely as follows: Values of 0.00 indicate poor agreement; 0.01–0.20 a slight agreement; 0.21–0.40 a fair agreement; 0.41–0.60 a moderate agreement; 0.61–0.80 a substantial agreement; and 0.81–1.00 an almost perfect agreement.

## 3. Results

### 3.1. Phase 1: Content and Ecological Validity of the Questionnaire

The initial version was assessed in phase one by the panel of experts, and the most relevant considerations and modifications in six dimensions were as follows: (a) general PA, improvement of the wording of some items and their order, dividing an item into two (due to complexity and subsections) and clarifying what was meant by PA and/or unorganised sport; (b) family and friendships, elimination of an where the question was included in another item; (c) spaces/facilities, modification of the question and answer options to include whether spaces and/or facilities were available for the practice of PAS and also what type they were; (d) motivation, change of name because it was originally called beliefs and did not reflect its content, oriented to the motives/reasons for the practice of PAS; (e) current PA state, change of response options because some of them were not exclusive; and (f) future, improvement of the wording of the items to differentiate them from each other.

The improved questionnaire, composed of twenty-five items and ten dimensions, was again reviewed and contrasted by the sport managers from the Gipuzkoa Provincial Council. Taking into account the contributions made by the experts in the validation process, the decision was made to eliminate Item 1 (information covered by the items that composed the general PA and transportation dimensions), Item 10 (non-relevant information) and Item 24 (covered by items 23 and 25). The elimination of this last item led to the unification of two dimensions (current and future state), because Item 23 collected information on both dimensions (Appendix C).

The comparison of the quantitative assessment of the experts is illustrated in Table 1. The results were statistically significant, increasing the total values in phase two (3.75 ± 0.29 vs. 3.95 ± 0.07; *p* < 0.05; η^2^ = 0.676; large; Δ% = 5.33) in comparison to phase 1. Considering the specific indicators, significant changes were also observed in phase 2 in comparison to phase 1 for the total values of sufficiency, clarity, coherence and relevance (*p* < 0.05; η^2^ > 0.287; large; Δ% > 2.34).

### 3.2. Phase 2: Reliability of the Questionnaire

The final questionnaire (twenty-two items, nine dimensions) was distributed to primary school students at two different times (T and R-T). The results of the reliability and agreement assessment are illustrated in Table 2. No significant differences were observed in seventeen of the twenty-two items; however, there were significant differences in the other five items (i.e., items 3, 4, 7, 10 and 18). On the other hand, significant agreement was observed in all items (Kappa range: 0.234–0.952, from regular agreement to almost perfect agreement, *p* < 0.05), except for Item 4 (Kappa = 0.022, slight agreement, *p* > 0.05).

## 4. Discussion

The goal of the study was to design, validate and determine the reliability of the Physical Activity and Sport Habits Questionnaire for Children from a multidimensional perspective to measure PAS habits in schoolchildren aged 8 to 12 years. The objective of the questionnaire developed in the study was to collect precise and complete information on PAS habits of students attending primary school in the province of Gipuzkoa, Spain, in order to facilitate the design of different strategies to promote active habits for different public administrations, in general, and the Gipuzkoa Provincial Council in particular. The multidimensional questionnaire was designed with a holistic vision of PAS habits from an ecological approach in order to create active living communities, guaranteeing measurement of key dimensions for understanding PAS habits as a whole including overall PA, organised sport and PA, active play, active transportation, sedentary behaviour, family and peers, community and environment, and difficulties and reason to practise PAS. As explained previously, even though there are valid questionnaires [53,58,59,60,62,63,64] that address some of the Global Matrix 4.0 indicators, no questionnaire has been found that assesses the majority of these relevant indicators at the same time when assessed in primary school students [61].

For this reason, the “Physical Activity and Sport Habits Questionnaire for Children” includes all the dimensions, studied separately in different questionnaires mentioned previously, so it can provide multidimensional information relevant for increasing the level of PAS practice and promoting active and healthy habits in children. At the same time, it can be sustainable over time due to the not excessively high number of items that comprise it.

The questionnaire initially designed was subjected to a validation and reliability process performed in two phases. In the first phase, as argued by García de Yébenes et al. [73], after having performed a review of the literature, twenty-five items organised into ten dimensions were selected to be part of the questionnaire. In addition, the content and ecological validity were assessed by a panel of experts. This panel was composed of seventeen experts who took part in this phase, a higher number than in other studies with a similar topic [74,75,76], and an appropriate number according to Escobar-Pérez and Cuervo-Martínez [69], who suggested a number between two and twenty experts.

These experts made both quantitative and qualitative contributions, a fundamental factor in the content validity process [75,77]. After taking into account their relevant contributions and indications [69], they observed a significant improvement of the questionnaire’s sufficiency, clarity, coherence and relevance. This improvement resulted from the adequacy and elimination of items based on qualitative assessments during the process of verifying content validity.

On the other hand, as performed in a previous study [78], ecological validity was ensured from a socio-ecological perspective through the participation of the sport managers from the Gipuzkoa Provincial Council, members specialised in the area of knowledge and in the characteristics of the physical and sociocultural environment of the study population [79]. Mainly, the use of the concept ‘multisport’ was qualified when referring to the school sport programme and, in turn, corresponded to the philosophy and guidelines of the Gipuzkoa Provincial Council programme. Along these same lines, the list of sports and/or physical activities performed or desired by children based on the offer of the province programme was completed.

At the same time, the response options of the individuals who influenced the students were carefully specified, as well as the options for existing barriers and motivations, i.e., relevant information for determining future sport policies and strategies.

In the second phase of this study, the sample size used was similar or larger, in the vast majority of cases, than samples assessed in other international and national studies in which the reliability of questionnaires on PA habits in children had been assessed [80,81]. Good reliability values were obtained in most of the items included in the questionnaire (absence of significant differences or regular–almost perfect agreement between the T and the R-T), although in eight items the reliability values were of slight agreement (Kappa < 0.4). These reliability results between the T and the R-T are similar to those obtained in other studies in which the items had exhibited a regular agreement or greater than K ≥ 0.31 in all its items [82,83,84]. It is worth noting that, in the questionnaire, significant differences were obtained between the T and the R-T in items 3, 4, 7b, 10b and 18a and b. These results coincide with those obtained in previous studies in which some items also had not exhibited adequate reliability values [52,85], even though the students were older than the schoolchildren assessed in the present study (≥13 years). The slight agreement in some items could be explained by several factors, such as the characteristics of the sample (children) [84], the number of response options of these items or the score prevalence. In addition, for example, Item 3 of the present questionnaire refers to the moderate practice of PA. Possibly, the term moderate may not be adequately identified by children and would require a clearer explanation in its formulation. Thus, low correlation values were observed in the item of PA with moderate intensity, and the objective values indicated a regular agreement with the current guidelines on PA [83]. Although Item 3 (moderate PAS practice 1 h/day) did not obtain adequate reliability values in the present questionnaire, it is a reference value to determine sedentary lifestyle [22], and it has been an item validated and used in various questionnaires and/or previous studies [45].

Possibly, the different results of the previous studies in comparison to the results of the present study could be due to the fact that the questionnaires previously used had been applied to children of older ages than the schoolchildren of the present study. This way, the use of practice times could make it difficult to properly understand that question by primary school students. On the other hand, Item 4 (not organised PAS) did not obtain an adequate reliability value either. Along the same lines, Cleland et al. [85] observed significant agreement in most of the items in a study assessing the reliability of the self-reported questionnaire of individual-level perceptions of the PA environment; however, they did not obtain adequate values in the item related to unorganised PAS in 18-year-old participants.

In this sense, Batista et al. [31] indicated that it was easier to remember organised practice, especially competitive practice, in comparison to unorganised practice, which could explain the difficulty of accurately reporting this type of PAS [86]. This aspect could have been one of the causes of the inadequate reliability values in this item. However, based on different studies, it has been described that knowing more about this type of unorganised practice is of vital importance [87,88]. This way, it could be interesting to reformulate the question and/or offer more information about the type of practice to the schoolchildren who complete the questionnaire.

With respect to Item 7b, referring to the situation regarding the programme, the fact that in some cases the respondents performed the R-T after a variable time from two weeks to two months could have caused changes in the situation regarding the sport programme or PAS practice, thus compromising reliability values. On the other hand, although items 10b (referring to the use of recreational screen time during the weekend) and 18a and b (hours of sleep during the week and weekend) have been validated in previous studies [39,49,51,57], the reliability values indicated regular–moderate agreements, being somewhat higher in the study conducted by Segura-Díaz et al. [84], who obtained moderate values of sedentary habits in children. Possibly the respondents did not adequately recognise the number of hours they spent on weekends with recreational screen time and the hours of sleep, so these items would require greater specification/reformulation or being contrasted with objective measurement methods.

Based on the results obtained in the present study, the researchers propose the use of the multidimensional twenty-two-item questionnaire organised into nine dimensions (Appendix A and Appendix B), although it is necessary to restructure some items and responses in order to improve T vs. R-T reliability. The number of items proposed in this questionnaire may be appropriate since, based on the panel of experts, it measures the construct of interest and does not fatigue or demotivate the participants [89].

### Limitations

Although the present study was performed with high methodological and ethical rigor, it was not free of limitations. The main limitation is that, between the T and the R-T, there was a variable separation from two weeks to two months during the academic year. This fact may have influenced the results and contributed to greater differences and lower agreement values in some items. This fact occurred because the recruitment and the distribution of questionnaires were performed in the schools, with their daily reality and capabilities. This way, neither the researchers nor the technicians of the Gipuzkoa Provincial Council had the competence to be able to accelerate the process, so that all students could respond with an interval of two weeks. The ecological nature of the study, specially intended to cover the needs of this province in the north of Spain, did not make it possible to have participants from other communities, regions or countries. Therefore, it is important to note that the results may not be generalised to other populations or contexts. Further studies are necessary to validate the questionnaire and verify its reliability in different samples and assess its applicability in other settings. Finally, a discriminant validation based on different constructs could not be performed. Thus, it would be interesting to delve into this aspect in further studies.

## 5. Conclusions

The results of the present study determined the validity and reliability of the Physical Activity and Sport Habits Questionnaire for Children as an appropriate instrument to measure PAS habits in schoolchildren aged eight to twelve years in the province of Gipuzkoa, Spain, in the two official languages of the territory, i.e., Basque and Spanish, although some items require additional explanation and further specification to enhance their reliability. Taking into account the results of content and ecological validity and reliability, the use of the version with twenty-two items and nine dimensions is proposed (Appendix A) until future studies can propose a revised version to enhance the reliability of these items. Its application will allow the investigation of the PAS habits of schoolchildren from a multidimensional perspective and thus the determination of different areas for improvement, as well as the design of effective interventions that promote more active and healthy lifestyles from an early age.

## Figures and Tables

**Table 1:** Quantitative assessment of the two review phases performed by the panel of experts, differential analysis and magnitude of change.

II/FI	Phase 1 Mean ± SD	Phase 2 Mean ± SD	Wilcoxon *p* Value	Effect Size (η^2^)	Percentage Change (Δ%)
1/1#	-	-	-	-	-
2/2	3.71 ± 0.52	3.96 ± 0.17	0.020 *	0.363	6.74
3/3	3.76 ± 0.41	3.87 ± 0.37	0.317	0.067	2.93
4/4	3.58 ± 0.67	3.93 ± 0.19	0.016 *	0.387	9.78
5/5	3.67 ± 0.52	4.00 ± 0.00	0.010 *	0.447	8.99
6/6	3.69 ± 0.56	4.00 ± 0.00	0.026 *	0.330	8.40
7/9	3.87 ± 0.25	3.96 ± 0.17	0.334	0.062	2.33
8/7	3.78 ± 0.33	3.96 ± 0.17	0.123	0.159	4.76
8/8	3.78 ± 0.33	3.89 ± 0.21	0.272	0.080	2.91
9/10#	-	-	-	-	-
10/11	3.82 ± 0.31	3.98 ± 0.09	0.066	0.226	4.19
11/12	3.80 ± 0.37	3.93 ± 0.19	0.157	0.133	3.42
12/13	3.89 ± 0.24	3.96 ± 0.12	0.257	0.086	1.80
13/14	3.73 ± 0.48	3.93 ± 0.19	0.111	0.169	5.36
14/15	3.73 ± 0.48	3.93 ± 0.14	0.084	0.198	5.36
15/16	3.93 ± 0.19	3.91 ± 0.15	0.739	0.007	−0.51
16/17	3.44 ± 0.88	3.91 ± 0.23	0.027 *	0.325	13.66
17/17	-	-	-	-	-
18/18	3.71 ± 0.45	4.00 ± 0.00	0.026 *	0.332	7.82
19/19	3.58 ± 0.60	3.98 ± 0.09	0.011 *	0.435	11.17
20/20	3.82 ± 0.33	3.93 ± 0.26	0.102	0.178	2.88
21/21	3.82 ± 0.45	3.98 ± 0.09	0.102	0.178	4.19
22/22	3.78 ± 0.39	3.98 ± 0.09	0.039 *	0.283	5.29
23/23	3.56 ± 1.04	3.98 ± 0.09	0.102	0.178	11.80
24/24#	-	-	-	-	-
25/25	3.84 ± 0.31	3.89 ± 0.21	0.750	0.007	1.30
Total S	3.51 ± 0.61	3.91 ± 0.24	0.038 *	0.287	11.40
Total Cl	3.65 ± 0.34	3.95 ± 0.07	0.001 *	0.676	8.22
Total Co	3.75 ± 0.42	3.96 ± 0.08	0.027 *	0.324	5.60
Total R	3.84 ± 0.21	3.93 ± 0.13	0.021 *	0.356	2.34
Total	3.75 ± 0.29	3.95 ± 0.07	0.001 *	0.676	5.33

II, initial item; FI, final item; η^2^, eta squared; #, item eliminated in the validation process; S, sufficiency; Cl, clarity; Co, coherence; R, relevance; * *p* < 0.05—significant differences between phase 1 and phase 2.

**Table 2:** Results of the reliability and agreement assessment (T and R-T) of the school-age children’s active habits questionnaire.

Item	McNemar, McN-Bowker or Wilcoxon *p* Value	Cohen’s Kappa	*p* Value
1. Type of S-PA practice	-	0.809	0.000
2. S-PA practice type desire	-	0.569	0.000
3. Moderate S-PA practice—1 h/day	0.002 *	0.511	<0.001
4. Unorganized S-PA	<0.001 *	0.022	0.411
5. Organized S-PA	0.607	0.372	<0.001
6. SS program participation	1.000	0.925	<0.001
7a. SS program evaluation	0.923	0.576	<0.001
7b. Situation regarding SS programme	0.042 *	0.594	<0.001
8. Participation in competitions	0.851	0.724	<0.001
9a. Home-school transfer	0.187	0.859	<0.002
9b. Transfer from school to home	0.070	0.886	<0.001
10a. Recreational screen time during the week	0.231	0.378	<0.001
10b. Weekend recreational screen time	0.038 *	0.492	<0.001
11. Practice of S-PA in the environment	0.537	0.952	0.000
12. Influence for practising S-PA	0.558	0.931	0.000
13. S-PA practice with family members	0.572	0.380	<0.001
14. S-PA practice with friends	0.211	0.410	<0.001
15. Encourage friends to practise S-PA	1.000	0.487	<0.001
16. Place near/home to practise S-PA	0.081	0.887	0.000
17. Nearby place/school to practise S-PA	0.346	0.854	0.000
18a. Hours of sleep during the week	0.037 *	0.316	<0.001
18b. Weekend sleep hours	0.010 *	0.388	<0.001
19. Difficulties practising S-PA	0.250	0.595	<0.001
20. Reasons to practise S-PA	0.922	0.911	0.000
21. Current S-PA situation/state	0.147	0.234	<0.001
22. Senior S-PA practice	0.453	0.352	<0.001

S-PA, sport and/or PA; SS, school sport; PE, physical education; * *p* < 0.05.

## Data Availability

The data presented in this study are available on request from the corresponding author due to legal and ethical reasons.

## Appendix A. Physical Activity and Sport Habits Questionnaire for Children (Basque and Spanish Version)

**Oharra: oso garrantzitsua da haurrek lasai eta modu espontaneoan erantzutea, ondo pentsatzeko denbora hartuz, baina erantzunak bilatu gabe./Nota:** es muy importante que los niños respondan con tranquilidad y de manera espontánea, tomándose su tiempo para pensar bien, pero sin rebuscar las respuestas.

**DATU OROKORRAK/DATOS GENERALES**

**Udalerria/Municipio**
(* Esta pregunta es obligatoria)

**Ikastetxea/Centro escolar**
(* Esta pregunta es obligatoria)

**Maila/Curso**
(* Esta pregunta es obligatoria)
(* Marque una sola opción)

☐ 3. maila/3° curso
☐ 4. maila/4° curso
☐ 5. maila/5° curso
☐ 6. maila/6° curso

**Generoa/Género**
(* Esta pregunta es obligatoria)
(* Marque una sola opción)

☐ neska/chica
☐ mutila/chico
☐ ez bitarra/no binario
☐ nahiago dut ez erantzun/prefiero no contestar

**GALDETEGIA/CUESTIONARIO**

1. **Zeintzuk dira gehien egiten dituzun kirol edota jarduera fisikoak? (gehienez 3 aukeratu)/¿Cuáles son los deportes y/o actividades físicas que más practicas? (elige máximo 3)**

(* Esta pregunta es obligatoria)

☐ Bat ere ez/Ninguno
  ☐ Aerobika/Aerobic
  ☐ Arku tiraketa/Tiro con arco
  ☐ Arrantza/Pesca
  ☐ Arraunketa/Remo
  ☐ Atletismoa/Atletismo
  ☐ Automobilismoa (karting)/Automovilismo (karting)
  ☐ Badmintona/Bádminton
  ☐ Beisbol eta sofbola/Béisbol y sófbol
  ☐ Bela/Vela
  ☐ Belar gaineko hockeya/Hockey sobre hierba
  ☐ Bodyboarda/Bodyboard
  ☐ Bola eta Toka/Bola y Toca
  ☐ Boleibola/Voleibol
  ☐ Dantzak/Bailes
  ☐ Futbola/Fútbol
  ☐ Esgrima/Esgrima
  ☐ Eskalada/Escalada
  ☐ Eskia/Esquí
  ☐ Eskubaloia/Balonmano
  ☐ Esku pilota/Pelota mano
  ☐ Gimnastika/Gimnasia
  ☐ Golfa/Golf
  ☐ Halterofilia/Halterofilia
  ☐ Herri Kirolak/Herri Kirolak
  ☐ Hipika/Hípica
  ☐ Igeriketa/Natación
  ☐ Igeriketa artistikoa/Natación artística
  ☐ Irristaketa/Patinaje
  ☐ Izotz gaineko irristaketa/Patinaje sobre hielo
  ☐ Izotz gaineko hockeya/Hockey sobre hielo
  ☐ Judoa/Judo
  ☐ Karatea/Karate
  ☐ Kirol egokitua/Deporte adaptado
  ☐ Mahai tenisa/Tenis de mesa
  ☐ Mendizaletasuna/Montañismo
  ☐ Padela/Pádel
  ☐ Paddel surfa/Paddel surf
  ☐ Parkour/Parkour
  ☐ Piraguismoa/Piragüismo
  ☐ Pool edo Karanbola Billarra/Billar Pool o Carambola
  ☐ Saskibaloia/Baloncesto
  ☐ Scooter/Scooter
  ☐ Skate/Skate
  ☐ Snowboard/Snowboard
  ☐ Sorospena/Socorrismo
  ☐ Squash/Squash
  ☐ Surfa/Surf
  ☐ Taekwondoa/Taekwondo
  ☐ Tenisa/Tenis
  ☐ Triatloia/Triatlon
  ☐ Txirrindularitza/Ciclismo
  ☐ Urpeko jarduerak/Actividades subacuáticas
  ☐ Waterpoloa/Waterpolo
  ☐ Windsurfa/Windsurf
  ☐ Xakea/Ajedrez
  ☐ Yoga/Yoga
  ☐ Zesta punta/Cesta punta
  ☐ Zirku jarduerak/Actividades circenses
  ☐ Besteak/Otros

2. **Egiten ez dituzunetatik, zein kirol edota jarduera fisiko egitea gustatuko litzaizuke? (gehienez 3 aukeratu)/De los que no practicas, ¿qué deporte y/o actividad física te gustaría practicar? (elige máximo 3)**

(* Esta pregunta es obligatoria)

☐ Bat ere ez gehiago/Ninguno más
  ☐ Aerobika/Aerobic
  ☐ Arku tiraketa/Tiro con arco
  ☐ Arrantza/Pesca
  ☐ Arraunketa/Remo
  ☐ Atletismoa/Atletismo
  ☐ Automobilismoa (karting)/Automovilismo (karting)
  ☐ Badmintona/Bádminton
  ☐ Beisbol eta sofbola/Béisbol y sófbol
  ☐ Bela/Vela
  ☐ Belar gaineko hockeya/Hockey sobre hierba
  ☐ Bodyboarda/Bodyboard
  ☐ Bola eta Toka/Bola y Toca
  ☐ Boleibola/Voleibol
  ☐ Dantzak/Bailes
  ☐ Futbola/Fútbol
  ☐ Esgrima/Esgrima
  ☐ Eskalada/Escalada
  ☐ Eskia/Esquí
  ☐ Eskubaloia/Balonmano
  ☐ Esku pilota/Pelota mano
  ☐ Gimnastika/Gimnasia
  ☐ Golfa/Golf
  ☐ Halterofilia/Halterofilia
  ☐ Herri Kirolak/Herri Kirolak
  ☐ Hipika/Hípica
  ☐ Igeriketa/Natación
  ☐ Igeriketa artistikoa/Natación artística
  ☐ Irristaketa/Patinaje
  ☐ Izotz gaineko irristaketa/Patinaje sobre hielo
  ☐ Izotz gaineko hockeya/Hockey sobre hielo
  ☐ Judoa/Judo
  ☐ Karatea/Karate
  ☐ Kirol egokitua/Deporte adaptado
  ☐ Mahai tenisa/Tenis de mesa
  ☐ Mendizaletasuna/Montañismo
  ☐ Padela/Pádel
  ☐ Paddel surfa/Paddel surf
  ☐ Parkour/Parkour
  ☐ Piraguismoa/Piragüismo
  ☐ Pool edo Karanbola Billarra/Billar Pool o Carambola
  ☐ Saskibaloia/Baloncesto
  ☐ Scooter/Scooter
  ☐ Skate/Skate
  ☐ Snowboard/Snowboard
  ☐ Squash/Squash
  ☐ Surfa/Surf
  ☐ Taekwondoa/Taekwondo
  ☐ Tenisa/Tenis
  ☐ Triatloia/Triatlon
  ☐ Txirrindularitza/Ciclismo
  ☐ Sorospena/Socorrismo
  ☐ Urpeko jarduerak/Actividades subacuáticas
  ☐ Waterpoloa/Waterpolo
  ☐ Windsurfa/Windsurf
  ☐ Xakea/Ajedrez
  ☐ Yoga/Yoga
  ☐ Zesta punta/Cesta punta
  ☐ Zirku jarduerak/Actividades circenses
  ☐ Besteak/Otros

3. **Egiten al duzu, gutxienez egunean ordu betez (60 minutuz), arnasa azkar edo zailtasunez hartzera behartzen zaituzten edota izerdia botarazten dizuten kirolik edota jarduera fisikorik?/¿Practicas deporte y/o actividad física, que te obliguen a respirar deprisa o con dificultad y/o te hagan sudar, al menos durante 1 hora (60 minutos) al día?**

(* Esta pregunta es obligatoria)
(* Marque una sola opción)

☐ Bai/Sí
  ☐ Ez/No

4. **Gorputz Hezkuntzako orduak kontuan hartu gabe, egiten duzu ANTOLATU GABEKO kirola edota jarduera fisikoa (zure denbora librean begirale edo entrenatzailerik gabe egiten dituzun jarduerak)?/Sin contar las horas de Educación Física, ¿haces deporte y/o actividad física NO ORGANIZADO (actividades que realizas en tu tiempo libre sin monitores/as o entrenadores/as)?**

(* Esta pregunta es obligatoria)
(* Marque una sola opción)

☐ Bai/Sí
  ☐ Ez/No

5. **Gorputz Hezkuntzako orduak kontuan hartu gabe, egiten duzu ANTOLATUTAKO kirola edota jarduera fisikoa (ikastetxean, klubean, kiroldegian, gimnasioan edo antzeko leku batean, begirale edo entrenatzailearekin egiten dituzun jarduerak)?/Sin contar las horas de Educación Física, ¿haces deporte y/o actividad física ORGANIZADO (aquellas actividades que realizas en el centro escolar, en un club, polideportivo, gimnasio o sitio parecido con monitor/a o entrenador/a)?**

(* Esta pregunta es obligatoria)
(* Marque una sola opción)

☐ Bai/Sí
  ☐ Ez/No

6. **Parte hartzen al duzu zure ikastetxeko eskola-kiroleko programan (multikirola)?/¿Participas en el programa de deporte escolar (multikirola) de tu centro escolar?**

(* Esta pregunta es obligatoria)
(* Marque una sola opción)

☐ Bai/Sí
  ☐ Ez/No

7A. **Baloratu ezazu, oro har, zure ikastetxeko eskola-kiroleko programa (multikirola)./Valora de una manera global el programa de deporte escolar (multikirola) de tu centro escolar.**

(* Esta pregunta es obligatoria)
(* Marque una sola opción)

(* Contestar solo si han contestado a “Parte hartzen al duzu zure ikastetxeko eskola-kiroleko programan (multikirola)?/¿Participas en el programa de deporte escolar (multikirola) de tu centro escolar?”: “Bai/Sí”)

☐ Txarra/Malo
  ☐ Erdipurdikoa/Regular
  ☐ Ona/Bueno
  ☐ Oso ona/Muy bueno
  ☐ Bikaina/Excelente

7B. **Adierazi zein den zure egoera eskola-kiroleko programarekiko (multikirola)/Indica cuál es tu situación respecto al programa de deporte escolar (multikirola).**

(* Esta pregunta es obligatoria)
(* Marque una sola opción)

(* Contestar solo si han contestado a “Parte hartzen al duzu zure ikastetxeko eskola-kiroleko programan (multikirola)?/¿Participas en el programa de deporte escolar (multikirola) de tu centro escolar?”: “Ez/No)

☐ Nire ikastetxean ez dago horrelako programarik/No existe dicho programa en mi centro escolar
  ☐ Parte hartu dut, baina utzi egin dut/He participado, pero lo he dejado
  ☐ Ez dut inoiz parte hartu/No he participado nunca

8. **Parte hartzen al duzu kirol lehiaketetan?/¿Participas en competiciones deportivas?**

(* Esta pregunta es obligatoria)
(* Marque una sola opción)

☐ Bai/Sí
  ☐ Ez/No

9. **Adierazi normalean nola joaten eta itzultzen zaren zure etxetik ikastetxera./Indica cómo vas y vuelves habitualmente desde tu casa al centro escolar.**

(* Esta pregunta es obligatoria)
(* Marque una sola opción por fila)

	Oinez/Andando	Bizikletaz, monopatinez, patinetez eta abarrez (motorrik gabe)/En bicicleta, monopatín, patinete, etc. (sin motor)	Autobusez, autoz, motorrez, tranbiaz, trenez, patinetez (motorrarekin)/En autobús, coche, moto, tranvía, tren, patinete (con motor)
Joanekoa/Ida	☐	☐	☐
Itzulerakoa/Vuelta	☐	☐	☐

10. **Zure denbora librean, normalean, adierazi zenbat ordu ematen dituzun egunean hurrengo jardueretan: sare sozialetan, telebista ikusten, ordenagailuan/tablet-ean/mugikorrean jolasten edota bideoak ikusten, kontsolekin jolasten, interneten nabigatzen, etab./En tu tiempo libre, habitualmente, indica cuántas horas dedicas al día a alguna de estas actividades: redes sociales, ver televisión, jugar y/o ver videos en el ordenador/tablet/móvil, jugar con consolas, navegar por internet, etc.**

(* Esta pregunta es obligatoria)
(* Marque una sola opción por fila)

	2 ordu baino gutxiago/menos de 2 horas	2 eta 4 ordu bitartean/entre 2 y 4 horas	4 ordu baino gehiago/más de 4 horas
Eskolako eguna/Día de escuela	☐	☐	☐
Jaieguna/Día festivo	☐	☐	☐

11. **Adierazi zure familian eta lagunartean, normalean kirola edota jarduera fisikoa egiten duten pertsona guztiak./Indica todas las personas que practican deporte y/o actividad física de forma habitual en tu entorno familiar y amistades.**

(* Esta pregunta es obligatoria)

☐ Ama/Madre
  ☐ Aita/Padre
  ☐ Anai-arrebak/Hermano/a
  ☐ Beste senitarteko batzuk/Otros familiares
  ☐ Lagunak/Amigos/as
  ☐ Inork ez/Nadie

12. **Adierazi kirola edota jarduera fisikoa egiteko zugan eragin positiboa duten pertsona guztiak./Indica todas las personas que influyen en ti positivamente para deporte y/o practicar actividad física.**

(* Esta pregunta es obligatoria)

☐ Ama/Madre
  ☐ Aita/Padre
  ☐ Anai-arrebak/Hermano/a
  ☐ Beste senitarteko batzuk/Otros familiares
  ☐ Lagunak/Amigos/as
  ☐ Gorputz Hezkuntzako irakaslea/Profesor/a de Educación Física
  ☐ Entrenatzailea/Entrenador/a
  ☐ Inork ez/Nadie

13. **Zenbatero egiten duzu kirola edota jarduera fisikoa zure familiarekin?/¿Con qué frecuencia practicas deporte y/o actividad física con tus familiares?**

(* Esta pregunta es obligatoria)
(* Marque una sola opción)

☐ Inoiz ez/Nunca
  ☐ Batzuetan/A veces
  ☐ Askotan/A menudo
  ☐ Beti/Siempre

14. **Zenbatero egiten duzu kirola edota jarduera fisikoa zure lagunekin?/¿Con qué frecuencia practicas deporte y/o actividad física con tus amigos/as?**

(* Esta pregunta es obligatoria)
(* Marque una sola opción)

☐ Inoiz ez/Nunca
  ☐ Batzuetan/A veces
  ☐ Askotan/A menudo
  ☐ Beti/Siempre

15. **Animatzen al dituzu lagunak kirola edota jarduera fisikoa egitera?/¿Animas a tus amigos/as para que practiquen deporte y/o actividad física?**

(* Esta pregunta es obligatoria)
(* Marque una sola opción)

☐ Bai/Sí
  ☐ Ez/No

16. **Ba al dago zure etxetik hurbil kirola edota jarduera fisikoa egiteko lekurik? (adierazi dituzun aukera guztiak)/¿Hay algún lugar cerca de tu casa para practicar deporte y/o actividad física? (indica todas las opciones que tienes)?**

(* Esta pregunta es obligatoria)

☐ Kiroldegia/Polideportivo
  ☐ Parkea/Parque
  ☐ Jolas eremua/Zona de juego
  ☐ Natur ingurunea/Medio natural
  ☐ Ez dago/No existe

17. **Ba al dago zure ikastetxetik hurbil kirola edota jarduera fisikoa egiteko lekurik? (adierazi dituzun aukera guztiak)/¿Hay algún lugar cerca de tu centro escolar para practicar deporte y/o actividad física? (indica todas las opciones que tienes)**

(* Esta pregunta es obligatoria)

☐ Kiroldegia/Polideportivo
  ☐ Parkea/Parque
  ☐ Jolas eremua/Zona de juego
  ☐ Natur ingurunea/Medio natural
  ☐ Ez dago/No existe

18. **Normalean, zenbat ordu egiten dituzu lo egunean?/Habitualmente, ¿cuántas horas duermes al día?**

(* Esta pregunta es obligatoria)
(* Marque una sola opción por fila)

	8 ordu baino gutxiago/menos de 8 horas	8 eta 10 ordu bitartean/entre 8 y 10 horas	10 ordu baino gehiago/más de 10 horas
Eskolako eguna/Día de escuela	☐	☐	☐
Jaieguna/Día festivo	☐	☐	☐

19. **Adierazi kirola edota jarduera fisikoa egiteko dituzun zailtasun guztiak/Indica todas las dificultades que tienes para practicar deporte y/o actividad física.**

(* Esta pregunta es obligatoria)

☐ Ez daukat oztoporik/No tengo barreras
  ☐ Ez zait gustatzen kirola edota jarduera fisikoa/No me gusta el deporte y/o la actividad física
  ☐ Ez naiz ona kiroletan edota jarduera fisikoetan/No soy bueno/a en los deportes y/o en las actividades físicas
  ☐ Ez zait gustatzen lehiatzea/No me gusta competir
  ☐ Oso nekatuta nago kirola edota jarduera fisikoa egiteko/Estoy muy cansado/a para hacer deporte y/o actividad física
  ☐ Etxeko lan gehiegi ditut edota asko ikasi behar dut/Tengo demasiados deberes y/o tengo que estudiar mucho
  ☐ Ez dut astirik/No tengo tiempo
  ☐ Diru asko balio du/Cuesta mucho dinero
  ☐ Osasun arrazoiengatik/Por motivos de salud
  ☐ Beste oztopo batzuk/Otras barreras

20. **Adierazi kirola edota jarduera fisikoa egiteko dituzun arrazoi guztiak./Indica todas las razones que tienes para practicar deporte y/o actividad física.**

(* Esta pregunta es obligatoria)

☐ Ez daukat arrazoirik/No tengo razones
  ☐ Lagunekin egoteko/Para estar con mis amigos/as
  ☐ Itxura fisikoa zaintzeko/Para cuidar mi aspecto físico
  ☐ Ongi pasatzeko eta gozatzeko/Para divertirme y disfrutar
  ☐ Lehiatzea gustatzen zaidalako/Porque me gusta competir
  ☐ Gurasoek esaten didatelako/Porque lo dicen mis madres/padres
  ☐ Osasungarria delako/Porque es saludable
  ☐ Beste arrazoi batzuengatik/Por otras razones

21. **Aukeratu zuri gehien egokitzen zaizun egoera./Elige la situación que más se ajuste a ti.**

(* Esta pregunta es obligatoria)
(* Marque una sola opción)

☐ Ez dut kirolik edota jarduera fisikorik egiten, eta ez dut egin nahi/No practico deporte y/o actividad física y no quiero practicarlo
  ☐ Ez dut kirolik edota jarduera fisikorik egiten, baina egin nahi dut/No practico deporte y/o actividad física, pero quiero practicarlo
  ☐ Kirola edota jarduera fisikoa aldizka egiten dut (astean hainbat aldiz) baina ez dut egiten jarraitu nahi/Practico deporte y/o actividad física regularmente (varias veces por semana) pero no quiero seguir practicándolo
  ☐ Kirola edota jarduera fisikoa aldizka egiten dut (astean hainbat aldiz) eta egiten jarraitu nahi dut/Practico deporte y/o actividad física regularmente (varias veces por semana) y quiero seguir practicándolo

22. **Zure ustez, heldua izatean, kirola edota jarduera fisikoa ohikotasunez egingo duzu?/¿Crees que, cuando seas mayor, practicarás deporte y/o actividad física de forma habitual?**

(* Esta pregunta es obligatoria)
(* Marque una sola opción)

☐ Bai/Sí
  ☐ Ez/No

## Appendix B. Physical Activity and Sport Habits Questionnaire for Children (English Version *)

**** (The questionnaire has not been validated in this language. Only for reading purposes)***

**GENERAL INFORMATION**

**Municipality**
(* This question is mandatory)

**School center**
(* This question is mandatory)

**Grade**
(* This question is mandatory)
(* Select only one option)

☐ 3rd grade (8–9 years old)
  ☐ 4th grade (9–10 years old)
  ☐ 5th grade (10–11 years old)
  ☐ 6th grade (11–12 years old)

**Gender**
(* This question is mandatory)
(* Select only one option)

☐ Female
  ☐ Male
  ☐ Non-binary
  ☐ Prefer not to answer

**QUESTIONNAIRE**

1. **What are the sports and/or physical activities that you practice the most? (choose up to 3)**

(* This question is mandatory)

☐ None
  ☐ Aerobics
  ☐ Archery
  ☐ Fishing
  ☐ Rowing
  ☐ Athletics
  ☐ Karting
  ☐ Badminton
  ☐ Baseball and Softball
  ☐ Sailing
  ☐ Field Hockey
  ☐ Bodyboarding
  ☐ Ball games
  ☐ Volleyball
  ☐ Dance
  ☐ Football
  ☐ Fencing
  ☐ Climbing
  ☐ Skiing
  ☐ Handball
  ☐ Basque pelota
  ☐ Gymnastics
  ☐ Golf
  ☐ Weightlifting
  ☐ Traditional Basque Sports
  ☐ Horseback riding
  ☐ Swimming
  ☐ Artistic Swimming
  ☐ Skating
  ☐ Ice Skating
  ☐ Ice Hockey
  ☐ Judo
  ☐ Karate
  ☐ Adapted sports
  ☐ Table tennis
  ☐ Mountaineering
  ☐ Padel tennis
  ☐ Paddel surfing
  ☐ Parkour
  ☐ Canoeing
  ☐ Billiards
  ☐ Basketball
  ☐ Scooter
  ☐ Skateboarding
  ☐ Snowboarding
  ☐ Lifesaving
  ☐ Squash
  ☐ Surfing
  ☐ Taekwondo
  ☐ Tennis
  ☐ Triathlon
  ☐ Cycling
  ☐ Underwater Activities
  ☐ Water polo
  ☐ Windsurfing
  ☐ Chess
  ☐ Yoga
  ☐ Jai-Alai
  ☐ Circus Activities
  ☐ Others

2. **From the ones you don’t practice, which sports or physical activities would you like to practice? (choose up to 3)**

(* This question is mandatory)

☐ None
  ☐ Aerobics
  ☐ Archery
  ☐ Fishing
  ☐ Rowing
  ☐ Athletics
  ☐ Karting
  ☐ Badminton
  ☐ Baseball and Softball
  ☐ Sailing
  ☐ Field Hockey
  ☐ Bodyboarding
  ☐ Ball games
  ☐ Volleyball
  ☐ Dance
  ☐ Football
  ☐ Fencing
  ☐ Climbing
  ☐ Skiing
  ☐ Handball
  ☐ Basque pelota
  ☐ Gymnastics
  ☐ Golf
  ☐ Weightlifting
  ☐ Traditional Basque Sports
  ☐ Horseback riding
  ☐ Swimming
  ☐ Artistic Swimming
  ☐ Skating
  ☐ Ice Skating
  ☐ Ice Hockey
  ☐ Judo
  ☐ Karate
  ☐ Adapted sports
  ☐ Table tennis
  ☐ Mountaineering
  ☐ Padel tennis
  ☐ Paddel surfing
  ☐ Parkour
  ☐ Canoeing
  ☐ Billiards
  ☐ Basketball
  ☐ Scooter
  ☐ Skateboarding
  ☐ Snowboarding
  ☐ Lifesaving
  ☐ Squash
  ☐ Surfing
  ☐ Taekwondo
  ☐ Tennis
  ☐ Triathlon
  ☐ Cycling
  ☐ Underwater Activities
  ☐ Water polo
  ☐ Windsurfing
  ☐ Chess
  ☐ Yoga
  ☐ Jai-Alai
  ☐ Circus Activities
  ☐ Others

3. **Do you practice sports or physical activities that require you to breathe quickly or with difficulty and/or make you sweat, for at least 1 h (60 min) per day?**

(* This question is mandatory)
  (* Select only one option)

☐ Yes
  ☐ No

4. **Apart from Physical Education hours, do you participate in NON-ORGANIZED sports or physical activities (activities you do in your free time without instructors or coaches)?**

(* This question is mandatory)
  (* Select only one option)

☐ Yes
  ☐ No

5. **Apart from Physical Education hours, do you participate in ORGANIZED sports or physical activities (activities you do in school, clubs, sports centers, gymnasiums, or similar places with an instructor or coach)?**

(* This question is mandatory)
  (* Select only one option)

☐ Yes
  ☐ No

6. **Do you participate in the school sports program (multisport) of your school?**

(* This question is mandatory)
  (* Select only one option)

☐ Yes
  ☐ No

7A. **Overall, rate your school sports program (multisport).**

(* This question is mandatory)
  (* Select only one option)
  (* Answer only if you answered “Yes” to “Do you participate in the school sports program (multisport) of your school?”)

☐ Poor
  ☐ Fair
  ☐ Good
  ☐ Very good
  ☐ Excellent

7B. **Indicate your situation regarding the school sports program (multisport).**

(* This question is mandatory)
  (* Select only one option)
  (* Answer only if you answered “No” to “Do you participate in the school sports program (multisport) of your school?”)

☐ There is no such program in my school
  ☐ I participated but dropped out
  ☐ I have never participated

8. **Do you participate in sports competitions?**

(* This question is mandatory)
  (* Select only one option)

☐ Yes
  ☐ No

9. **Indicate how you usually go to and from your home to school.**

(* This question is mandatory)
  (* Select only one option per row)

	On foot	Cycling, skateboarding, scootering (non-motorized)	By bus, car, motorcycle, tram, train, scooter (with motor)
Going to school	☐	☐	☐
Returning from school	☐	☐	☐

10. **In your free time, usually, indicate how many hours you spend per day on the following activities: social networks, watching television, playing and/or watching videos on the computer/tablet/mobile, playing with consoles, browsing the internet, etc.**

(* This question is mandatory)
  (* Select only one option per row)

	Less than 2 h	Between 2 and 4 h	More than 4 h
Schoolday	☐	☐	☐
Weekend/Holiday	☐	☐	☐

11. **Indicate all the people who regularly practice sports and/or physical activities in your family and friends.**

(* This question is mandatory)

☐ Mother
  ☐ Father
  ☐ Siblings
  ☐ Other relatives
  ☐ Friends
  ☐ No one

12. **Indicate all the people who positively influence you to practice sports and/or physical activities.**

(* This question is mandatory)

☐ Mother
  ☐ Father
  ☐ Siblings
  ☐ Other relatives
  ☐ Friends
  ☐ Physical Education teacher
  ☐ Coach
  ☐ No one

13. **How often do you practice sports and/or physical activities with your family?**

(* This question is mandatory)
  (* Select only one option)

☐ Never
  ☐ Sometimes
  ☐ Often
  ☐ Always

14. **How often do you practice sports and/or physical activities with your friends?**

(* This question is mandatory)
  (* Select only one option)

☐ Never
  ☐ Sometimes
  ☐ Often
  ☐ Always

15. **Do you encourage your friends to practice sports and/or physical activities?**

(* This question is mandatory)
  (* Select only one option)

☐ Yes
  ☐ No

16. **Is there a place near your home to practice sports and/or physical activities? (indicate all options)**

(* This question is mandatory)

☐ Sports center
  ☐ Park
  ☐ Playground
  ☐ Natural environment
  ☐ None

17. **Is there a place near your school to practice sports and/or physical activities? (indicate all options)**

(* This question is mandatory)

☐ Sports center
  ☐ Park
  ☐ Playground
  ☐ Natural environment
  ☐ None

18. **Usually, how many hours do you sleep per day?**

(* This question is mandatory)
  (* Select only one option per row)

	Less than 8 h	Between 8 and 10 h	More than 10 h
Schoolday	☐	☐	☐
Weekend/Holiday	☐	☐	☐

19. **Indicate all the difficulties you have for practicing sports and/or physical activity.**

(* This question is mandatory)

☐ I have no barriers
  ☐ I don’t like sports and/or physical activity
  ☐ I’m not good at sports and/or physical activities
  ☐ I don’t like to compete
  ☐ I’m too tired to do sports and/or physical activity
  ☐ I have too much homework and/or I have to study a lot
  ☐ I don’t have time
  ☐ It costs too much money
  ☐ For health reasons
  ☐ Other barriers

20. **Indicate all the reasons you have for practicing sports and/or physical activity.**

(* This question is mandatory)

☐ I have no reasons
  ☐ To be with my friends
  ☐ To take care of my physical appearance
  ☐ To have fun
  ☐ Because I like to compete
  ☐ Because my parents say so
  ☐ Because it’s healthy
  ☐ Other reasons

21. **Choose the situation that best fits you.**

(* This question is mandatory)
  (* Select only one option)

☐ I don’t practice sports and/or physical activity, and I don’t want to
  ☐ I don’t practice sports and/or physical activity, but I want to
  ☐ I practice sports and/or physical activity occasionally (several times a week) but I don’t want to continue
  ☐ I practice sports and/or physical activity occasionally (several times a week) and I want to continue

22. **Do you think, when you are older, you will regularly practice sports and/or physical activity?**

(* This question is mandatory)
  (* Select only one option)

☐ Yes
  ☐ No

## Appendix C. Items Eliminated After the Validation Process of the Initially Designed Physical Activity and Sport Habits Questionnaire for Children

1. **Normalean egiten al duzu kirola edota jarduera fisikorik?/¿Practicas deporte y/o actividad física de forma habitual?/*Do you practice sports and/or physical activity regularly?***

(* Esta pregunta es obligatoria/*This question is mandatory*)
(* Marque una sola opción/*Select only one option*)

☐ Bai/Sí/*Yes*
  ☐ Ez/No/*No*

10. **Gorputz Hezkuntza irakasgaiko zenbat ordu dituzu astean?/¿Cuántas horas a la semana tienes la asignatura de Educación Física?/*How many hours a week do you take Physical Education?***

(* Esta pregunta es obligatoria/*This question is mandatory*)
(* Marque una sola opción/*Select only one option*)

☐ Astean ordu 1 baino gutxiago/Menos de 1 hora a la semana/*Less than 1 h a week*
  ☐ Astean ordu 1 eta 2 artean/Entre 1 y 2 horas a la semana/*Between 1 and 2 h a week*
  ☐ Astean 2 ordu baino gehiago/Más de 2 horas a la semana/*More than 2 h a week*

24. **Zure ustez, datorren ikasturtean kirola edota jarduera fisikoa ohikotasunez egingo duzu?//¿Crees que, el próximo curso, practicarás deporte y/o actividad física de forma habitual?/*Do you think that next year you will practice sports and/or physical activity on a regular basis?***

(* Esta pregunta es obligatoria)/*This question is mandatory*)
(* Marque una sola opción/*Select only one option*)

☐ Bai/Sí/*Yes*
  ☐ Ez/No/*No*
